# A Food-Based Intervention in a Military Dining Facility Improves Blood Fatty Acid Profile

**DOI:** 10.3390/nu14040743

**Published:** 2022-02-10

**Authors:** Asma S. Bukhari, Laura J. Lutz, Tracey J. Smith, Adrienne Hatch-McChesney, Kristie L. O’Connor, Christopher T. Carrigan, Michael R. Hawes, Susan M. McGraw, Kathryn M. Taylor, Catherine M. Champagne, Scott J. Montain

**Affiliations:** 1 Military Nutrition Division of the US Army Research Institute of Environmental Medicine, Natick, MA 01760, USA; laura.j.lutz2.civ@mail.mil (L.J.L.); tracey.smith10.civ@mail.mil (T.J.S.); Adrienne.M.McChesney.civ@mail.mil (A.H.-M.); kloihst@rit.edu (K.L.O.); christopher.t.carrigan.civ@mail.mil (C.T.C.); susan.m.mcgraw6.civ@mail.mil (S.M.M.); smontain@gmail.com (S.J.M.); 2 Belovo Inc., Southern Pines, NC 28387, USA; belovo@earthlink.net; 3 Military Performance Division of the US Army Research Institute of Environmental Medicine, Natick, MA 01760, USA; Kathryn.M.Taylor41.civ@mail.mil; 4 Pennington Biomedical Research Center, Louisiana State University, Baton Rouge, LA 70808, USA; Catherine.Champagne@pbrc.edu

**Keywords:** omega-3 fatty acid, omega-6 fatty acid, lipid profile, dining facility, omega-3 index

## Abstract

Enhancing dietary omega-3 highly unsaturated fatty acids (n-3 HUFA) intake may confer neuroprotection, brain resiliency, improve wound healing and promote cardiovascular health. This study determined the efficacy of substituting a few common foods (chicken meat, chicken sausage, eggs, salad dressings, pasta sauces, cooking oil, mayonnaise, and peanut butter) lower in omega-6 polyunsaturated fatty acids (n-6 PUFA) and higher in n-3 HUFA in a dining facility on blood fatty acid profile. An eight-week prospective, between-subjects (*n* = 77), repeated measures, parallel-arm trial was conducted. Participants self-selected foods consumed from conventionally produced foods (control), or those lower n-6 PUFA and higher n-3 HUFA versions (intervention). Changes in blood omega-3 index, eicosapentaenoic acid (EPA), docosahexaenoic acid (DHA), n-6 PUFA, lipid profile, and food satisfaction were main outcomes. Between-group differences over time were assessed using a linear mixed model to measure the effect of diet on blood serum fatty acids and inflammatory markers. The intervention group achieved a higher omega-3 index score (3.66 ± 0.71 vs. 2.95 ± 0.77; *p* < 0.05), lower total n-6 (10.1 ± 4.6 vs. 15.3 ± 6.7 µg/mL; *p* < 0.05), and higher serum concentration of EPA (5.0 ± 1.31 vs. 4.05 ± 1.56 µg/mL; *p* < 0.05) vs. controls. Satisfaction in intervention foods improved or remained consistent. Substitution of commonly eaten dining facility foods with like-items higher in DHA and EPA and lower in n-6 PUFA can favorably impact fatty acid status and the omega-3 index.

## 1. Introduction

The fatty acid composition of typical U.S. diets has changed drastically over the past century, due to changes in food production practices, fish intake, and displacement of animal fat oils with plant-based oils [1,2]. The shift to plant-based, notably soybean sources, as feed for both animals and humans, has simultaneously reduced saturated fat intake and increased intake of omega-6 polyunsaturated fatty acids (n-6 PUFAs). From 1909 to 1999, the per capita consumption of poultry increased by 454% and soybean use by >1000 fold [1]. As a consequence, the typical American diet has shifted from approximately 1% energy to more than 8% energy from n-6 PUFAs [1]. Findings from the National Health and Nutrition Examination Survey (NHANES 2013–2016) regarding usual nutrient intake of n-3 and n-6 PUFA from food and beverages of adults (19–50 years) indicate above adequate intake (AI), of linoleic acid (LA) and alpha linolenic acid (ALA), but eicosapentaenoic acid (EPA) and docosahexaenoic acid (DHA) [3] consumption were less than the minimum recommendation of 250 mg/day each [4]. The European Food Safety Authority recommends 250–500 mg of EPA and DHA per day from the diet to reduce cardiovascular risks among European adults [5].

The n-3 and n-6 PUFAs are important in a variety of physiological functions, however, their effects are opposing in nature [6]. A favorable n-3:n-6 PUFA status is associated with lowered heart disease risk, improved immune function and wound healing, and lowered anxiety and suicidal thoughts [7,8,9,10,11]. The military has recognized the potential health benefits from optimal dietary fatty acid composition [12,13,14], and studies have suggested that n-3 and n-6 PUFA levels in the body can influence cognition and psychological health of military personnel [15,16]. A recent publication reviewed the role of nutrition and global burden of infection and highlighted omega-3 fatty acid supplementation as a safe, effective, and a low-cost strategy to support the immune system [17]. In addition, optimal fatty acid composition and ratios appear to assist with prevention and treatment of obesity and cancer [18]. Data from US military suicide deaths identified low n-3 HUFA status in military personnel and suggested a need for well-designed intervention trials [19].

The n-3 and n-6 PUFAs are essential dietary fatty acids that cannot be synthesized by the human body, but only interconverted on a limited basis and must be obtained through diet or supplementation [7,20]. The n-3 PUFAs include ALA, EPA, and DHA. Alpha-linolenic acid is found in plant-based foods such as flaxseed, walnuts, and canola oil, and the human body has limited capability to convert ALA to EPA and DHA. Both EPA and DHA are primarily found in animal-based foods and found in abundance in fatty fish. The n-6 PUFAs include LA and arachidonic acid (AA), and are found in abundance in soybean, corn, sunflower, safflower, cotton seed oils [20].

Government and professional organizations recommend increasing consumption of the highly unsaturated n-3 fatty acids (n-3 HUFA), EPA and DHA, through food or supplementation [7,21,22]. Based on NHANES 2013–2016 data, 20.1% of Americans met the recommendation of consuming seafood twice/week, a decline from the previous NHANES survey [23], indicating challenges with incorporating seafood in the diets of Americans. The concentration of n-3 HUFA in marine food sources varies considerably and depends on wild or farmed conditions and feeding practices [24]. Use of dietary supplements is another approach to improve n-3 HUFA intake. However, increasing n-3 HUFA intake by consuming oily fish and regular dietary supplementation does not remediate the excess n-6 PUFA in the diet. A fatty acid supplementation study using krill oil in military personnel failed to show treatment effect on outcomes due to poor compliance to n-3 pills [25,26].

Fortifying or formulating commonly consumed foods to contain higher n-3 HUFA and lower n-6 PUFA is an alternative approach for improving fatty acid status [27,28]. Emerging agricultural and food production technologies have enabled production of oilseeds and animal feeds that are lower in n-6 PUFAs and higher in n-3 HUFAs [29,30], resulting in commercially available vegetable oils, animal meats and eggs with a more favorable fatty acid profile. These products enable commonly consumed foods (i.e., margarine, eggs) to be used to increase n-3 HUFA intake without changing eating habits [31].

The current study is a follow-up to the study by Young et al. who performed a 10-week placebo-controlled, double-blind laboratory-based feeding study and found consumption of specially produced, low n-6 and high n-3 PUFA foods improved plasma and red blood cell PUFA status and induced robust increases in the n-3 index [27]. This study determined whether a similar effect could be replicated in an ad libitum dining hall style, eating environment. Since the earlier study by Young et al. demonstrated the effectiveness of this food-based approach. Every effort was made to mimic the usual eating environment to include limited or no nutrition education on the intervention foods. The experimental food items were simply incorporated by direct food-item swapping. The study also utilized low-burden data collection methods to capture frequency and selection of the foods manipulated or swapped to mimic the usual eating environment. Conventional foods provided in a dining hall were exchanged with like alternatives containing higher n-3 HUFA and lower n-6 PUFA. Whether participants would voluntarily choose these foods frequently enough, and in sufficient quantity, to produce meaningful improvements in blood fatty acid profiles was the primary outcome of interest.

## 2. Materials and Methods

### 2.1. Participants and Study Design

This prospective, between-subjects, repeated measures, parallel arm study was conducted at the Natick Soldier Systems Center (NSSC) dining facility (DFAC) from 2014 to 2016. This study was approved by the Human Use Review Committee at the US Army Research Institute of Environmental Medicine and all participants provided written informed consent. The study design and methods were consistent with the goal of testing the intervention in a naturalistic setting. The NSSC DFAC is a traditional-style cafeteria that offers approximately three-four main line entrees per meal. This DFAC was selected due to size, accessibility to participants and ease of manipulating and monitoring food procurement and food production. Eligible participants were adults (90 M; 22 F) with access to the DFAC; all participants provided informed consent. Subsequently, 20 participants were withdrawn due to conflicting schedules and 16 withdrew for other reasons, leaving 77 study completers (63 M; 14 F).

During the control study phase, the DFAC prepared and served conventional foods using usual ingredients and procedures. In the intervention phase, certain foods were substituted for like items (chicken meat, chicken sausage, eggs, salad dressings, pasta sauces, cooking oil, mayonnaise, and peanut butter) having lower n-6 PUFA and higher n-3 HUFA levels compared to the conventional food items (refer to Supplemental Table S1 for fatty acid content of intervention and control foods). The DFAC staff used these foods/ingredients without changes to the menu, recipes or cooking procedures. Participants were not randomized because the food in the entire dining facility was affected during the study period. Therefore, they only completed one arm of the study (*n* = 39 control arm and *n* = 38 intervention arm). All dining facility patrons were exposed to the study diets (control and intervention), regardless of whether or not they were enrolled in the study.

After the study briefing and consent process, participants underwent baseline testing, which consisted of a demographic and background survey, measurement of height and body weight, completion of a food frequency questionnaire (FFQ), and a fasting blood sample to assess fatty acids, lipid profile, homocysteine, and high sensitivity C-reactive protein (hsCRP). Participants were instructed to avoid changes in their nutritional supplementation habits for the duration of the study and were required to consume at least two meals per day, five days per week, at the DFAC during the eight-week study. The number of meals consumed and foods selected were monitored weekly to reinforce the aforementioned instructions. Although the study was not blinded, participants were unaware of the change between control and intervention foods.

The intervention food items were produced from United States Department of Agriculture (USDA) approved sources. The anticipated dietary intake of the fatty acids that were enhanced in the intervention foods, EPA+DHA, were within recommended intake levels and at levels deemed generally recognized as safe (GRAS) by governmental agencies [5]. No health risks were identified in association with the intervention foods. At the end of the study period, participants completed another FFQ to capture habitual eating during the eight weeks. Body weight was measured, blood sampling occurred, and a DFAC satisfaction survey was completed.

### 2.2. Study Foods

#### 2.2.1. The Control Group

The control group foods were those normally served in dining hall. Prior to conduct of the study, the investigators extensively reviewed the menu for type of foods served, cooking procedures, and usage before identifying food items to be swapped. The study DFAC menu indicated offering over 166 foods and of these 37 (~22%) were food options which could be targeted (these included chicken-based items, eggs, sausage, pasta sauce, salad dressings, peanut butter mayonnaise and cooking oil). The production of the selected items was then coordinated with the study food supplier to ensure the foods produced and packaged appeared as similar as possible to the standard food items and for the timely delivery for frozen and non-frozen items such as eggs. The control food items were produced using the standard USDA specifications and supplied by existing food procurement contracts at the DFAC. The study investigators were not involved in procurement of these foods. The types of foods selected by study participants was monitored using meal tracking forms. Food production data were collected throughout the study period to track type and amount of food prepared and served in the DFAC. Supplementary Table S2 shows the typical layout of food items in DFAC and items served/swapped at breakfast, lunch and dinner meal. At breakfast, meal items identified to be swapped were eggs and recipes with eggs (for example, pancakes, French toast, omelets), sausage patties, peanut butter, and cooking oil. At lunch and dinner, one entrée was to be swapped of the three to four entrees served on a regular basis, and was either a chicken-based item or those using pasta sauce (for example, pasta entrée and pizza). Pasta sauces, salad dressings, peanut butter, and cooking oil were swapped for lunch and dinner.

#### 2.2.2. Intervention Group Foods

The intervention group foods consisted of eggs, chicken meat, chicken sausages, pasta sauces, salad dressings, peanut butter, mayonnaise, and cooking oil. The eggs and meats were produced using improved animal feeding ingredients while adhering to current commercial practices and food production strategies to improve n3 to n6 ratios. They were similar in portion and type to the control versions in order to eliminate confusion with kitchen staff during food preparation, and to mask the intervention so that ad libitum food choices would not be affected by the food alterations. The PUFA levels in the chicken meats varied depending on the presence or absence of skin and whether the meat was composed of white or dark meat (Supplementary Table S1). Likewise, the long chain fatty acid (LCFA) composition of the chicken items was dependent on how the meat was prepared (the control and intervention breaded chicken items were prepared with regular vegetable oils or high oleic acid oils, respectively). The condiments were produced by ingredient substitution.

The intervention foods provided to the DFAC were coordinated by Belovo Incorporated, Southern Pines NC and utilized a commercial USDA-approved supplier that met the USDA health and safety standards for production, handling, and shipping in a facility that was inspected and certified by the installation’s military food inspector. The intervention food supplier partners with leading commercial food companies and many of these food items are also available commercially at various supermarkets. The chicken and egg products were evaluated for taste and cooking acceptability by the Human Systems Integration and Sciences Division, U.S. Army Combat Capabilities Development Command-Soldier Center, Natick, MA, and received positive ratings. These same intervention foods were also used in a previous randomized controlled study [25] and were independently analyzed for nutritional content by Lipid Technologies, LLC, Austin, MN.

### 2.3. Measurements

#### 2.3.1. Demographic/Background Survey

Information on sex, ethnic and racial background, education, military status, body weight changes, number of meals typically consumed in a military dining facility, food allergies, use of dietary supplements, and adherence to any specialized diet was collected.

#### 2.3.2. Anthropometric Measurements

Body weight was measured to the nearest 0.1 kg at baseline and at the end of the eight-week study period in a fasted state, using a calibrated digital scale, with participants dressed in shorts and a t-shirt. Height was measured at baseline using a portable stadiometer to the nearest 0.1 cm. Body mass index (BMI) was calculated at baseline and at the end of the study period to assess for change [32].

#### 2.3.3. Dietary Intake and Meals Tracking

A combination of tools was utilized to assess the habitual nutrient take of study participants, food production by food-service staff and number of meals consumed at the study location and types of foods selected. Habitual food intake was assessed using a validated food frequency questionnaire (FFQ, Nutrition Quest, 2014) [33]. The FFQ was completed at baseline (to measure habitual intake over the previous six months) and after eight weeks of study participation (to measure habitual intake only during the past eight-week study period). Since this was a follow-up study, a more rigorous food intake data collection tool was not utilized. The intent was to assess types of foods selected based on self-reported data. Data generated from the FFQ included nutrient intake and diet quality. Diet quality was measured using the 2010 Healthy Eating Index (HEI), which measures adherence to the Dietary Guidelines for Americans [21,34]. Study investigators provided Nutrition Quest with the nutrient composition of the experimental foods for proper tabulation of nutrients consumed during the intervention arm. The intent of FFQ was to assess the array of food choices, and if these choices resulted in changes in dietary fatty acid intake by offering foods with enhanced fatty acid composition and without educating or informing the study participants.

A critical component of this study was to closely monitor the frequency that the select menu foods were chosen by the study participants. Two weekly checklists were used to track meals consumed at the DFAC (Supplementary Table S3). To ensure compliance of meal tracking and to accurately collect data on foods consumed, study staff met with the participants one-three times per week for review. The checklists asked about a variety of food items consumed in the DFAC, not just the intervention foods, so that participants would not suspect or potentially bias themselves towards selecting more or less of the intervention foods in question (Supplementary Table S4). Investigators worked closely with the DFAC staff during both control and intervention phases in tracking food production information to assess the amount of daily food prepared, sold, and left over. Copies of order forms and production documents were obtained and reviewed by study staff throughout the study. This assured consistency of menu offerings during both phases.

#### 2.3.4. Blood Sampling

Blood serum and plasma samples were obtained pre and post study. Samples were centrifuged, frozen, and stored until analysis at the Pennington Biomedical Research Center (PBRC, Baton Rouge, LA, USA). Lipids were measured on the Beckman Coulter DXC 600 Pro (Brea, CA, USA). Specifically, total cholesterol was measured enzymatically following binding with a specific anti human ß-lipoprotein antibody to bind to lipoproteins (Low Density Lipoprotein (LDL), Very-Low-Density Lipoprotein (VLDL), and chylomicrons) other than High Density Lipoprotein (HDL). Triglycerides were measured enzymatically using a glycerol blank. Low-density lipoprotein was calculated using the Friedewald equation. The hsCRP was measured using a solid-phase, chemiluminescent immunometric assay (Siemens Immulite 2000, Llanberis, UK). Fatty acids in red blood cells were measured using gas chromatography mass spectrometry (Agilent 5975, Santa Clara, CA, USA) after being separated from the plasma, repeatedly washed, and lysed by freezing and thawing. The omega-3 index was calculated using the sum of the amounts of erythrocyte fatty acids EPA and DHA expressed as percent of total erythrocyte fatty acids. Omega-3 index is used, clinically, as a potential risk factor for coronary heart disease [35]. Homocysteine was measured via an immunoassay with chemiluminescent (Siemens Immulite 2000, Llanberis, UK) detection from serum.

#### 2.3.5. Satisfaction Survey

Satisfaction of the overall quality of meals/foods consumed at the DFAC was assessed once at the end of the eight-week study period. The survey utilized a Likert scale (ratings 1–5), with one being strongly agree and five, strongly disagree. For each item, a five-point scale was used, with lower scores indicating higher satisfaction levels. This survey was previously used in our research [36].

### 2.4. Sample Size

The primary study outcome measures were changes in blood levels of n-3 and n-6 PUFA. To enable detection of a 10% change in plasma fatty acid with a standard deviation of 5% using a power of 80% and *p* < 0.05, approximately 15 subjects per group were required. That effect size (10% change) is smaller than that observed in a previous 10-week randomized controlled trial using similar food items [27]. In the previous trial, plasma n-6 PUFA decreased from 83 to 65% with a standard deviation (SD) of 5% [27]. Due to the variability expected in food preferences between individuals, the ad libitum food environment, and the presence of multiple food choices per meal, 45 volunteers per group were deemed necessary.

### 2.5. Statistical Analyses

Statistical analysis was performed using the SPSS statistical package (SPSS Inc., Chicago, IL, USA) version 23 [37]. Demographic variables, consumption of the study food items, and habitual dietary intake were compared between control and intervention groups using independent t-tests and chi-square analyses. A *p*-value of less than 0.05 was considered statistically significant. In order to evaluate the effect of the dietary intervention on anthropometric measures, tissue fatty acids and CVD risk factors, individual linear mixed models were used. The independent variables in the model were assignment in the intervention groups and time. The impact of the dietary intervention was evaluated with a group × time interaction within the model. Post-hoc *t*-tests were run for all models using Bonferroni adjusted *p*-values in order to account for multiple comparisons. All models were evaluated for homogeneity of variance and normality of residuals, which is a required assumption when using a linear model. In models where these assumptions did not hold, the dependent variable was transformed in order to make the assumptions hold. Within the analysis, the following variables were log-transformed: total fat, saturated fat, trans fat, monosaturated fat, polyunsaturated fat, linoleic (18:2, n-6), α-linolenic (18:3; n-3), total n-6, total n-3, C20:3 n-3, C18:2c Linoleic, C18.3 n-6 GLA, C20:4 AA, C22:6 DHA, Alternate-HEI, hsCRP, and triglycerides. The following variables were transformed using a cube root: total calories and stearidonic acid (18:4; n-3). All results are presented with the untransformed mean ± standard deviation of the variable evaluated.

## 3. Results

### 3.1. Participant Demographic and Anthropometric Measures

Demographic and anthropometric measures at baseline and after the eight-week study period are shown in Table 1. Body weight and BMI were not different between groups at either time point; however, both groups increased over time (*p* < 0.05).

### 3.2. Dietary Intake and Meal Tracking Outcomes

The number of control and intervention food servings consumed by the two eight-week study arms are presented in Table 2. Both groups achieved compliance with the required minimum number of weekly meals consumed at the DFAC, with no group differences in the number of meals consumed between the control (mean ± SD; 104 ± 23 meals) and intervention (98 ± 21) groups.

More chicken options were available for the control group and were selected more often by this group when compared to the intervention group. In both groups, eggs were the most frequently selected intervention food item, followed in order by chicken, sausage, salad dressing, pasta sauce, mayonnaise, and the least selected food item was peanut butter (Table 2).

The FFQ data (Table 3) revealed the intervention group, when compared to control, consumed higher daily amounts of EPA (mean ± SD; 0.115 ± 0.068 vs. 0.028 ± 0.034 g; *p* < 0.05), DPA (0.042 ± 0.027 vs. 0.012 ± 0.015 g; *p* < 0.05), and DHA (0.246 ± 0.129 vs. 0.06 ± 0.044 g; *p* < 0.05) during the study. In contrast there was a reduction in total n-6 (10.1 ± 4.6 vs. 15.3 ± 6.7 g; *p* < 0.05).

### 3.3. Biochemical Analysis

Blood triglycerides, HDL and hsCRP, were similar between groups at baseline and did not change over time in either group (Table 4). Total cholesterol, while similar at baseline between groups, increased over time in the intervention group by 5% (*p* = 0.01). There was no group × time interaction for HDL, LDL, triglycerides, homocysteine, or hsCRP. The red blood cell (RBC) fatty acids C14:0, C16:0, C16:1, C18:0, C18:1c, C20:0, C20:1, C20:2, C22:0, C22:1, C22:2, C24:0, C24:1 (data not shown), and C18:3 n-6, C18:3 n-3, C20:3 n-6 (Table 4) were similar between groups at baseline and did not change over time. After eight weeks, RBC C20:4 (AA) values were lower in the intervention group compared to the control group (mean ± SD; 205.6 ± 28.1 µg/mL vs. 264.2 ± 101.6 µg/mL; *p* = 0.003), but there were no significant changes within either group nor was there a significant group x time interaction. Unlike the control group, the intervention group saw increases in RBC C20:5 (EPA) concentration (3.26 ± 0.96 µg/mL to 5.00 ± 1.31 µg/mL; *p* = 0.003 group x time interaction) and reported significantly higher levels at the end of the study period. The intervention group also saw an increase in C22:6 (DHA) concentration (36.37 ± 9.80 µg/mL to 45.34 ± 9.19 µg/mL; *p* = 0.005 time effect) during the study period. However these differences did not result in a significant interaction for group x time. The RBC omega-3 index (EPA + DHA) improved for the intervention (2.83 ± 0.74 to 3.66 ± 0.71; *p* ≤ 0.05) and control groups (2.78 ± 0.80 to 2.95 ± 0.77; *p* <0.01); however, the intervention group had a significantly higher omega-3 index (average increase of 29%) at week eight compared to the control group (average increase of 6% increase) (*p* = 0.002 group effect).

### 3.4. Satisfaction towards DFAC Meals

There was no difference between control and intervention groups in their satisfaction in food appearance (control group 2.0 ± 1.1 vs. intervention group 1.9 ± 0.8), food taste (2.1 ± 1.1 vs. 1.9 ± 0.9, respectively), perception of availability of healthy food options (2.4 ± 1.3 vs. 2.0 ± 1.2, respectively), and overall acceptability of foods (2.3 ± 1.3 vs.1.8 ± 1.0, respectively).

## 4. Discussion

The current study assessed the effectiveness of a food-based strategy of modifying items consumed in ad libitum setting on enhancing the fatty acid profile of participants consuming those foods. Primarily, omega-3 index and blood fatty acid profiles were improved by consumption of eggs, chicken meat, chicken sausage, pasta sauce, salad dressings, and condiments that contained higher n-3 HUFA and lower n-6 PUFA compared to conventional alternatives. Manipulating the n-3 HUFA and n-6 PUFA ratio of commonly eaten food items contributed to the levels consumed during an eight-week period and subsequent alteration in blood fatty acid profiles. Modification of these foods did not result in a decline in meal satisfaction.

The magnitude of the blood fatty acid response observed in this study was less pronounced than that observed by Young et al. [27], probably because the previous study tightly controlled meal consumption on a daily basis. Young et al. required participants to consume 21 meals per week vs. the current study only required 10 meals per week, thus demonstrating correcting lipid balance is dose dependent on the number of meals with upgraded foods consumed per week. For example, Young et al. reported that red blood cell linoleic and arachidonic acid concentrations declined 13% and 7% while DHA and EPA increased 48% and 125%, respectively, when the diet provided ~0.9 g of HUFA per day [27]. In contrast, this study produced 6% and 5% reductions in linoleic and arachidonic acid concentrations while DHA and EPA rose 24% and 53%, respectively, when the diet provided ~0.4 g of HUFA per day. The average EPA intake of study participants at baseline was comparable to what was observed in the NHANES 2013–2016 report [3] and improved to levels above the 95th percentile of the general US population. The average DHA levels consumed by the study participants at baseline was similar to the national average and significantly increased by over 8-fold in the intervention group.

The number of non-intervention foods offered in the DFAC very likely contributed to the smaller EPA and DHA shift compared to Young et al. [27], and the varying menu schedule may have been a secondary contributing factor. For example, on a typical day, only 20–30% of the menu options contained the intervention food items. Individual food preferences also contributed to the magnitude of shift; while several of the intervention food items were selected frequently, others were not. Eggs, chicken meat and chicken sausage products were the main intervention contributors to the diet and the subsequent fatty acid profile shift observed. The limited use of salad dressings and condiment items was unexpected. A priori, it was assumed that salad dressing consumption would be much higher than it actually was, and a larger contributor to daily dietary EPA and DHA intake within the intervention group. The relatively limited use of these products within the study population calls into question the relative effectiveness of relying on margarine and vegetable oils to improve EPA and DHA status. The increase in total blood cholesterol in the intervention groups is somewhat surprising. The reason for this increase is unclear, as FFQ data did not reveal increased saturated fat or total fat intake relative to the control group, and blood triglycerides were not impacted. The previous study that manipulated the long-chain fatty acids of the study diets using similar intervention foods as used in the current study did not observe significant changes in fasting total cholesterol, LDL or HDL at any time or between intervention and control groups [27]. Likewise, a review of 49 RCTs on increasing PUFA intake and CVD risk factors showed slight reduction in triglycerides with little to no effect on total cholesterol, HDL-C or LDL-C. In contrast, a 12-week fish-oil supplementation study showed a significant increase in LDL-C and total cholesterol at 6 and 12 weeks in response to a 4 g/d dose of DHA and no significant group x time interaction for HDL-C [38]. An unexplained 5 to 10% and up to 45% increase in LDL-C was noted in review articles with multiple randomized controlled trials in response to prescription omega-3 preparations [11,39]. Of note, the 5% increase in total cholesterol observed in the current study falls within the known ~7% intra-individual variability in the measure, so may be entirely independent of the intervention [39].

The participants in this study initiated the intervention with the high n-6 and low n-3 status anticipated from consuming an American diet. The low status is consistent with other studies of military personnel. A cross-sectional study by Johnston et al., on omega-3 levels and neurocognitive performance among service members with mild-moderate depression had an average omega-3 index of 3.5 ± −0.7% (range 1.7 to 5.7%), with direct association between cognitive flexibility and executive function [40]. Lewis et al., reported presence of lower n-3 HUFA among US military personnel in a study examining relationship between serum DHA and suicide [19]. The NHANES data, too, reveal lower levels of long-chain omega-3 fatty acid among young adults (20–55 years) compared to seniors (>55 years) with 76% of young adults with less than 2.49% EPA+DHA [41]. These data illustrate that n-3 status is being negatively impacted by the current food source ecosystem and the dietary choices being made in this environment.

This study is not without limitations. Detailed dietary intake information regarding meals and snacks consumed outside the DFAC was not collected. FFQ was a useful tool for capturing the foods and approximate frequency and amounts consumed [42]. However, the FFQ is a self-report tool and therefore lacks precision in terms of calories consumed. The FFQ is able, however, to detect directional changes in the amount and type of fatty acid in a volunteer’s self-reported diet. The intent was to capture both habitual food intake regardless of location of meals consumed, the volunteer’s usage of dining facility and their selection of the experimental foods of interest while reducing subject burden and selection bias. Study strengths include testing the intervention in a naturalistic setting in a large sample consuming their meals in an ad libitum dining environment, and the inclusion of a control group. The study also showed feasibility of swapping certain food items with like items without negatively impacting both food preparation, service and food selection by study participants. That approach enables these findings to reflect the impact of individual food preferences and dietary habits on the efficacy of this study’s approach. Most notably, the use of commonly consumed food items as the vehicle to improve nutritional quality and the implementation of the intervention foods in the DFAC demonstrated an ad libitum intervention can successfully augment fatty acid intake.

## 5. Conclusions

Substitution of commonly eaten dining hall food items with like items formulated with higher levels of DHA and EPA and lesser amounts of n-6 PUFA is a feasible approach and can favorably impact fatty acid status and the omega-3 index. Eggs are a key food item, given their versatility, frequency of consumption in U.S. diets, and because DHA and EPA can be augmented quite dramatically without adversely affecting taste. Additional research should determine whether publicizing the higher n-3 HUFA and lower n-6 PUFA food substitutions to dining hall patrons would promote greater consumption of those items, thereby having an effect on lipid profile and inflammatory biomarkers. The implication of this study is that it highlights the viability of food-based approach to enhance omega-3 fatty acids in healthy individuals since compliance to supplements is suboptimal. Improving omega-3 fatty acids intake may help with neuroprotection, enhance injury recovery, and reduce depression and suicidal ideation. Future research should consider use of nutrition education along with tasty and appealing food options in cafeteria dining avenues to enhance meal quality.

## Figures and Tables

**Table 1:** Demographic and anthropometric measures of 77 participants in an 8-week parallel arms trial assessing the effects of dietary omega-3 fatty acids on blood lipid profiles ^a^.

Variable	Total	Control (*n* = 39)	Intervention (*n* = 38)	*p*-Value ^b^
Age (y) ^c^	25 ± 10	25 ± 10	24 ± 10	0.76
Height (cm) ^c^	175 ± 8	173 ± 6	177 ± 9	0.05
Weight (kg) ^c^				0.85
Baseline	81.2 ± 21.5	81.5 ± 22.3	80.9 ± 20.8	
Week 8	82.6 ± 21.5 ^d^	82.2 ± 21.9 ^d^	82.9 ± 21.3 ^d^	
Body mass index (kg/m^2^) ^c^				0.84
Baseline	26.4 ± 5.8	27.1 ± 6.2	25.8 ± 5.4	
Week 8	26.9 ± 5.8 ^d^	27.3 ± 6.0 ^d^	26.4 ± 5.5 ^d^	
Sex, *n* (%)			X^2^ (1) = 0.93, *p* = 0.38	
Male	64 (83)	34 (87)	30 (79)	
Female	13 (17)	5 (13)	8 (21)	
Race, *n* (%)			X^2^ (2) = 2.72, *p* = 0.26	
White/Caucasian	40 (52)	22 (56)	18 (47)	
Black/African American	20 (26)	7 (18)	13 (34)	
Other ^e^	17 (22)	10 (26)	7 (18)	
Ethnicity, *n* (%) ^f^			X^2^ (1) = 0.24, *p* = 0.81	
Non-Hispanic/Latino	52 (68)	25 (64)	27 (71)	
Hispanic	24 (31)	13 (33)	11 (29)	

^a^ In both control and intervention groups one volunteer completed the study at 6 weeks and two at 7 weeks; ^b^ Differences between groups were assessed using Student’s *t*-test for variables measured at baseline only; effects of time and group for weight and body mass index were determined by mixed model analysis; differences in categorical variables were determined using X^2^ test; ^c^ Continuous variables expressed as mean ± standard deviation; ^d^ Effect of time (*p* < 0.05); ^e^ Includes American Indian/Alaskan Native, Asian, Native Hawaiian/Pacific Islander, or Other when race was selected; ^f^ Missing data for one volunteer.

**Table 2:** Consumption of intervention foods at baseline and at the end of an 8-week parallel arms trial assessing the effects of dietary omega-3 fatty acids on blood lipid profiles in 77 participants ^a^.

Food, Serving Size	Control Group ^b^ (*n* = 39)	Intervention Group ^b^ (*n* = 38)	*p*-Value ^c^
Eggs, 1 whole	58 ± 22	55 ± 26	0.61
Frequency offered	Daily (B); 31% of the days (L or D)	Daily (B); 34% of the days (L or D)	
Chicken, 3 ounces	46 ± 18	34 ± 14	0.001
Frequency offered	3.3 options daily	2.9 options daily	
Sausage, 1 link	16 ± 17	12 ± 13	0.22
Frequency offered	Daily (B)	Daily (B)	
Salad Dressing, 1.5 oz	13 ± 15	13 ± 15	0.93
Frequency offered	Daily (L and D)	Daily (L and D)	
Pasta Sauce, 3 oz	9 ± 9	10 ± 9	0.71
Frequency offered	Daily (L and D)	Daily (L and D)	
Mayonnaise, 1 Tbsp	4 ± 8; 1 (0–33)	6 ± 8; 3 (0–40)	0.42
Frequency offered	Daily (L and D)	Daily (L and D)	
Peanut Butter, 0.75 oz	3 ± 8	3 ± 10	0.98
Frequency offered	Daily (L and D)	Daily (L and D)	

^a^ In both control and intervention groups one volunteer completed the study at 6 weeks and two at 7 weeks; ^b^ Values are mean ± standard deviation; ^c^ Between group differences determined by Student’s *t*-test. B = Breakfast; L = Lunch; D = Dinner.

**Table 3:** Dietary intake measured by food frequency questionnaire at baseline and at the end of an 8-week parallel arms trial assessing the effects of dietary omega-3 fatty acids on blood lipid profiles in 77 participants ^a^.

Nutrient	Control Group ^b^ (*n* = 39)	Intervention Group ^b^ (*n* = 38)	*p*-Value ^c^ Group x Time Interaction
Total calories, kcal			0.236
Baseline	2220 ± 899	2248 ± 829	
Week 8	2009 ± 842	1726 ± 714 *	
Total fat, g			0.325
Baseline	93.7 ± 39.5	91.2 ± 35.0	
Week 8	84.9 ± 41.2	72.5 ± 32.3 *	
Saturated fat, g			0.302
Baseline	30.5 ± 12.8	29.7 ± 11.7	
Week 8	28.4 ± 14.7	23.8 ± 11.5 *	
Monounsaturated fat, g			0.367
Baseline	36.9 ± 16.1	35.7 ± 13.4	
Week 8	33.1 ± 16.4	28.5 ± 13.0 *	
Polyunsaturated fat, g			0.237
Baseline	18.0 ± 8.3	17.9 ± 7.9	
Week 8	15.8 ± 7.5	13.4 ± 5.6 *	
Trans fat, g			0.325
Baseline	3.3 ± 1.7	3.3 ± 1.8	
Week 8	2.9 ± 1.8	2.5 ± 1.2 *	
Linoleic acid (18:2; n-6), g			0.0760
Baseline	15.3 ± 7.1	15.0 ± 6.6	
Week 8	13.2 ± 6.4	10.2 ± 4.6 *^†^	
α-linolenic acid (18:3; n-3), g			0.605
Baseline	1.47 ± 0.74	1.56 ± 0.97	
Week 8	1.30 ± 0.67	1.47 ± 0.78	
Stearidonic acid (18:4; n-3), g			0.0.911
Baseline	0.0032 ± 0.0025	0.0040 ± 0.0045	
Week 8	0.0038 ± 0.0038	0.0044 ± 0.0064	
Arachidonic acid (20:4; n-6), g			0.163
Baseline	0.20 ± 0.08	0.19 ± 0.09	
Week 8	0.23 ± 0.10	0.18 ± 0.09 ^†^	
EPA ^d^ (20:5; n-3), g			<0.001
Baseline	0.024 ± 0.018	0.028 ± 0.034	
Week 8	0.028 ± 0.027	0.115 ± 0.068 *^†^	
DPA ^e^ (22:5; n-3), g			<0.001
Baseline	0.011 ± 0.008	0.012 ± 0.015	
Week 8	0.014 ± 0.012	0.042 ± 0.027 *^†^	
DHA ^f^ (22:6; n-3), g			<0.001
Baseline	0.057 ± 0.032	0.060 ± 0.044	
Week 8	0.069 ± 0.043	0.246 ± 0.129 *^†^	
Total n-6, g			0.049
Baseline	15.5 ± 7.1	15.3 ± 6.7	
Week 8	13.4 ± 6.5	10.1 ± 4.6 *^†^	
Total n-3, g			0.0.072
Baseline	1.6 ± 0.8	1.7 ± 1.0	
Week 8	1.4 ± 0.7	1.9 ± 0.9 *	
HEI-2010 ^g^			0.827
Baseline	57.9 ± 10.1	55.9 ± 12.9	
Week 8	55.7 ± 9.9	52.9 ± 11.4	
n-6:n-3 ratio			<0.001
Baseline	10.2 ± 2.1	9.7 ± 1.9	
Week 8	10.1 ± 2.2	6.3 ± 3.8 *^†^	

^a^ In both the control and intervention groups one volunteer completed the study at 6 weeks and two at 7 weeks; ^b^ Values are mean ± standard deviation; ^c^ Group x Time Interaction; effects of time and group determined by mixed model analysis; ^d^ EPA = eicosapentaenoic acid; ^e^ DPA = docosapentaenoic acid; ^f^ DHA = docosahexaenoic acid; ^g^ HEI-2010 = Healthy Eating Index 2010 total score.* Effect of time, different from baseline (*p* < 0.05). ^†^ Effect of group, different from control (*p* < 0.05).

**Table 4:** Lipid profile, inflammatory biomarkers, and red blood cell fatty acids at baseline and end of an 8-week parallel arms trial assessing the effects of dietary omega-3 fatty acids on blood lipid profiles in 77 participants ^a^.

Analyte	Control Group ^b^ (*n* = 39)	Intervention Group ^b^ (*n* = 38)	*p*-Value ^c^ Group × Time Interaction
Total Cholesterol (mg/dL)			0.252
Baseline	164.8 ± 35.8	168.1 ± 32.4	
Week 8	161.5 ± 33.0	176.8 ± 34.6 ^†^	
High density lipoprotein (mg/dL)			0.928
Baseline	52.5 ± 11.2	50.6 ± 11.6	
Week 8	52.5 ± 11.8	50.5 ± 10.7	
Low density lipoprotein (mg/dL)			0.352
Baseline	90.0 ± 32.6	96.1 ± 31.7	
Week 8	92.8 ± 29.0	107.7 ± 31.0 ^†^	
Triglycerides (mg/dL)			0.212
Baseline	102.6 ± 96.1	98.1 ± 93.8	
Week 8	81.3 ± 49.2	92.8 ± 58.5	
hsCRP ^d^ (mg/L)			0.786
Baseline	2.75 ± 5.39	1.13 ± 1.75	
Week 8	2.67 ± 5.80	1.21 ± 2.00	
Homocysteine (µmol/L)			0.859
Baseline	7.13 ± 1.67	8.12 ± 1.50 ^†^	
Week 8	7.27 ± 1.95	8.40 ± 1.64 ^†^	
C18:3 n-3 ALA ^e^ (µg/mL)			0.873
Baseline	1.66 ± 0.80	1.58 ± 0.39	
Week 8	1.62 ± 0.69	1.50 ± 0.41	
C20:3 n-3 ETE ^f^ (µg/mL)			0.299
Baseline	1.04 ± 0.51	1.42 ± 0.35 ^†^	
Week 8	1.17 ± 0.49	1.44 ± 0.32 ^†^	
C20:5 n-3 EPA ^g^ (µg/mL)			0.003
Baseline	3.95 ± 2.43	3.26 ± 0.96	
Week 8	4.05 ± 1.56	5.00 ± 1.31 *^†^	
C18:3 n-6 GLA ^h^ (µg/mL)			0.873
Baseline	0.60 ± 0.33	0.45 ± 0.19 ^†^	
Week 8	0.70 ± 0.42	0.48 ± 0.16 ^†^	
C22:6 DHA ^i^ (µg/mL)			0.247
Baseline	40.56 ± 24.52	36.37 ± 9.8	
Week 8	41.81 ± 16.3	45.34 ± 9.1 ^†^	
C20:3 n-6 DHGLA ^j^ (µg/mL)			0.489
Baseline	22.73 ± 9.89	20.55 ± 4.60	
Week 8	23.58 ± 10.31	20.59 ± 5.02	
C18:2 Linoleic (µg/mL)			0.210
Baseline	167.21 ± 68.47	156.72 ± 25.90	
Week 8	170.73 ± 66.49	143.44 ± 23.26	
C20:4 AA ^k^ (µg/mL)			0.208
Baseline	250.50 ± 105.35	215.96 ± 38.78	
Week 8	264.23 ± 101.56	205.60 ± 28.1 ^†^	
Omega-3 index (EPA + DHA)			0.008
Baseline	2.78 ± 0.80	2.83 ± 0.74	
Week 8	2.95 ± 0.77	3.66 ± 0.71 *^†^	

^a^ In both the control and intervention groups one volunteer completed the study at 6 weeks and two at 7 weeks; ^b^ Values are mean ± standard deviation; ^c^ Group x time Interaction; effect determined by mixed model analysis; ^d^ hsCRP = high sensitivity C reactive protein; ^e^ ALA = alpha-linolenic acid; ^f^ ETE = eicosatrienoic acid; ^g^ EPA = eicosapentaenoic acid; ^h^ GLA = gamma-linolenic acid; ^I^ DHA = docosahexaenoic acid; ^j^ DHGLA = dihomo-gamma-linolenic acid; ^k^ AA = arachidonic acid. * Effect of time, different from baseline (*p* < 0.05). ^†^ Effect of group, different from control (*p* < 0.05).

## Data Availability

The data presented in this study are available on request from the corresponding author and if approval by the regulatory authority.

## Supplementary Materials

The following supporting information can be downloaded at: https://www.mdpi.com/article/10.3390/nu14040743/s1, Table S1: Fatty acid content of intervention foods compared with the USDA nutrient database offered during an 8-week parallel arms trial assessing the effects of dietary omega-3 fatty acids on blood lipid profiles. Table S2: Meal layout and food items offered and swapped. Table S3: Study participant meals tracking form. Table S4: Study participant food selection form.
