# Supplementary material for: A Food-Based Intervention in a Military Dining Facility Improves Blood Fatty Acid Profile
Source: Nutrients. 2022 Feb 10;14(4):743. doi: 10.3390/nu14040743 (PMC8879849; doi:10.3390/nu14040743)
Supplement: Supplementary file 1 [file nutrients-14-00743-s001.zip › nutrients-1561915-supplementary.pdf]

nutrients

MDPI

Supplementary Table S1. Fatty acid content of intervention foods compared with the USDA nutrient database offered during an 8-week parallel arms trial assessing the effects of dietary omega-3 fatty acids on blood lipid profiles.

Food item	Serving Amount	Total PUFA[a]	Total n-3	Total n-6	Total n-9	LA:ALA ratio	LA[b]	ALA[c]	EPA[d]	DPA[e]	DHA[f]	long chain n-3	n-6:n-3 ratio
		mg/serving											
**Chicken, whole, raw**	100g												
Control[g]		3230.0	190	2880.0	5170.0	23.1	3230.0	140.0	10.0	10.0	30.0	50.0	15.2
Intervention[h]		2266.0	799.5	1466.0	3119.0	1.9	1386.0	719.9	12.5	24.9	17.6	64.7	1.8
**Chicken, white, raw (+ skin)**	100g												
Control		1960.0	120.0	1790.0	3170.0	21.8	1740.0	80.0	10.0	10.0	20.0	40.0	14.9
Intervention		1101.5	385.6	715.9	1488.4	2.0	662.1	331.0	8.4	20.2	13.2	47.4	1.9
**Chicken, white, raw (- skin)**	100g												
Control		424.0	26.0	370.0	5830.0	24.4	317.0	13.0	3.0	6.0	4.0	13.0	14.2
Intervention		632.5	221.2	411.3	754.0	2.2	358.4	164.5	8.8	24.5	16.6	53.6	1.9
**Chicken, dark, raw (+ skin)**	100g												
Control		3105.7	155.7	2819.7	5307.0	20.0	2745.3	137.0	3.7	10.3	4.7	18.7	18.1
Intervention		2954.3	1048.6	1905.7	4078.7	1.9	1811.1	954.4	15.1	27.4	19.7	74.6	1.8
**Chicken, dark raw (- skin)**	100g												
Control		951.0	55.5	706	1225.0	20.5	676.0	33.0	3.0	9.5	10.0	22.5	12.7

Intervention		2234.2	791.8	1442.4	2887.0	1.9	1336.2	689.2	17.5	33.1	26.9	88.6	1.8
**Chicken nuggets**	100g												
Control		5486.5	348.0	5118.0	5906.0	14.8	5063.0	343.0	1.5	2.0	1.5	5.0	14.7
Intervention		7494.0	1059.5	6434.5	3331.6	6.3	6365.3	1008.4	11.2	18.8	15.4	50.0	6.0
**Cooked chicken, dark meat**	100g												
Control		3760.0	231.7	3387.7	6108.7	18.6	3368.7	181.0	8.0	17.3	25.3	50.7	14.6
Intervention		5143.4	1441.9	3701.5	4832.2	2.7	3609.5	1331.2	24.2	39.1	18.2	95.3	2.6
**Chicken Sausage**	100g												
Control		3560.0	310.0	3250.0	4570.0	12.4	3100.0	250.0	0	10.0	0	10.0	10.5
Intervention		8506.5	3349.1	5157.4	11103.4	1.6	4914.2	3054.1	62.5	96.0	46.2	204.7	1.5
**Whole egg, raw (2 eggs)**	100g												
Control		1911.0	110.0	1740.0	3411.0	43.2	1555.0	36.0	0.0	7.0	58.0	65.0	15.8
Intervention		2016.1	642.7	1373.4	3176.3	4.8	1237.9	257.7	56.0	39.8	274.5	370.3	2.1
**Salad Dressings**	30g												
Control		5648.3	580.1	5038.3	2372.7	8.7	5038.3	579.3	0.2	0.0	0.7	0.8	8.7
Intervention		1651.5	347.7	1303.8	6764.1	6.3	1291.1	201.8	46.9	7.1	69.7	128.8	3.7
**Mayonnaise**	15g												
Control		5120.0	580.0	5120.0	3100.0	8.8	5130.0	580.0	0.0	0.0	0.0	0.0	8.8
Intervention		2621.4	350.0	2271.4	6544.0	10.1	2255.3	223.9	39.9	5.9	60.2	110.7	6.5
**Peanut Butter**	32g												
Control		3630.0	16.0	3610.0	6360.0	300.8	3610.0	12.0	4.0	0.0	0.0	4.0	225.6
Intervention		2437.3	820.8	1616.5	10375.3	2.2	1608.8	734.6	35.4	4.4	31.0	75.5	2.0

**Pasta sauce**	62g												
Control		8369.1	941.5	7362.0	7031.9	7.8	7362.0	941.0	0.5	0.0	0.0	0.5	7.8
Intervention		7153.9	2792.3	4361.5	7938.3	2.0	4312.9	2143.6	205.3	35.0	304.1	567.7	1.6
**Cooking Oil**	100g												
Control		46075	5316	40758	33755	7.7	41159	5316	0	0	0	0	7.7
Intervention		8770	1950	6820	75460	3.5	6820	1952	0	0	0	0	3.5

PUFA = polyunsaturated fatty acid. [b]LA = Linoleic acid. [c]ALA = Alpha-linolenic acid. [d]EPA = Eicosapentaenoic acid. [e]DPA = Docosapentaenoic acid. [f]Docosahexaenoic acid. [g]Control foods, nutrient composition obtained from USDA database. Cooking oil fatty acid were estimated based on food labels and USDA database. [h]Intervention foods (except cooking oil), nutrient composition analyzed by an independent laboratory. Intervention cooking oil nutrient compostion is based on the manufactures fatty acid profile report.

Supplementary Table S2. Meal layout and food items offered and swapped.

Figure 2.1. Breakfast food selections with arrows pointing to the specific foods substituted or impacted by the substitution.

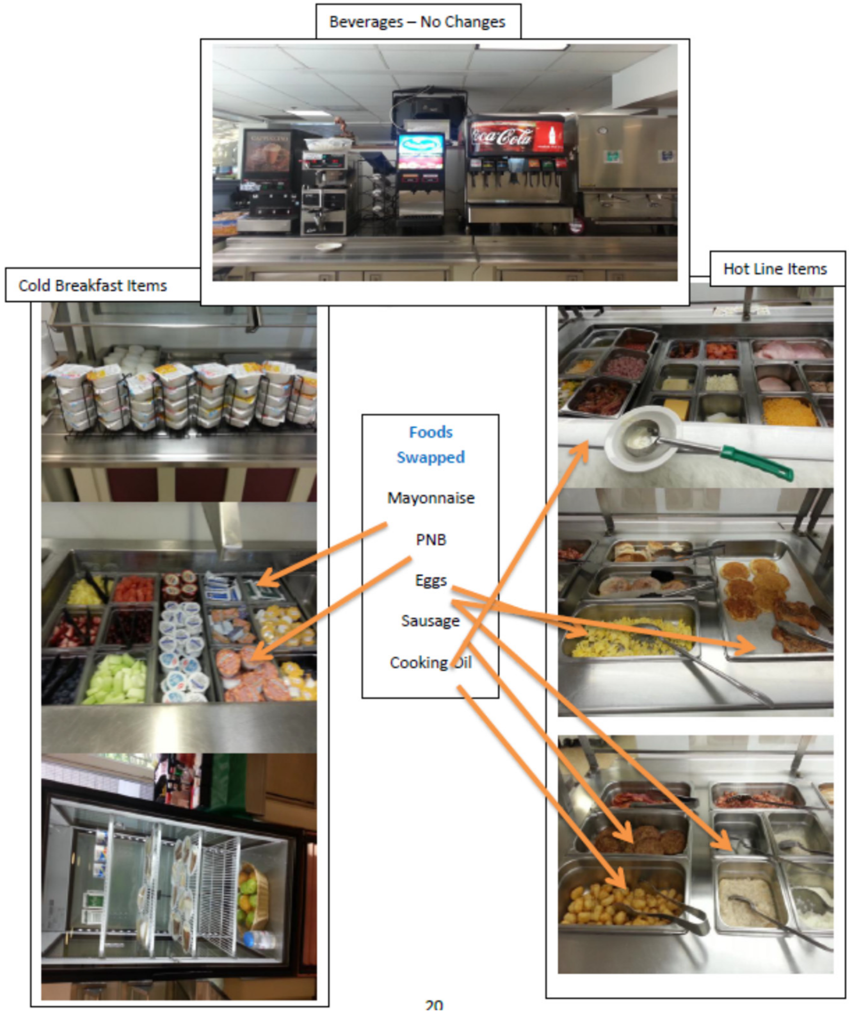

PNB= Peanut Butter

Figure 2.2. Lunch food selections with arrows pointing to the specific foods substituted or impacted by the substitution

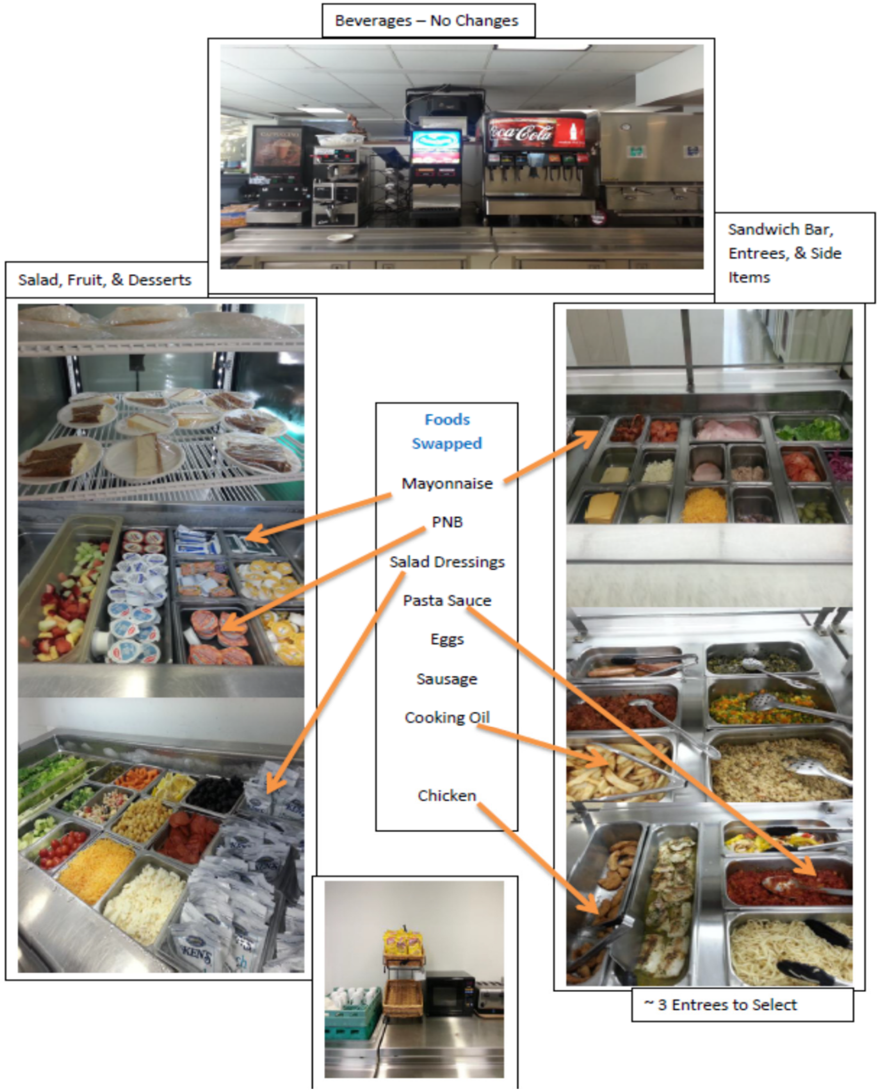

PNB= Peanut Butter

Figure 2.3. Dinner Selections with arrows pointing to the foods substituted or foods impacted by the food substitution

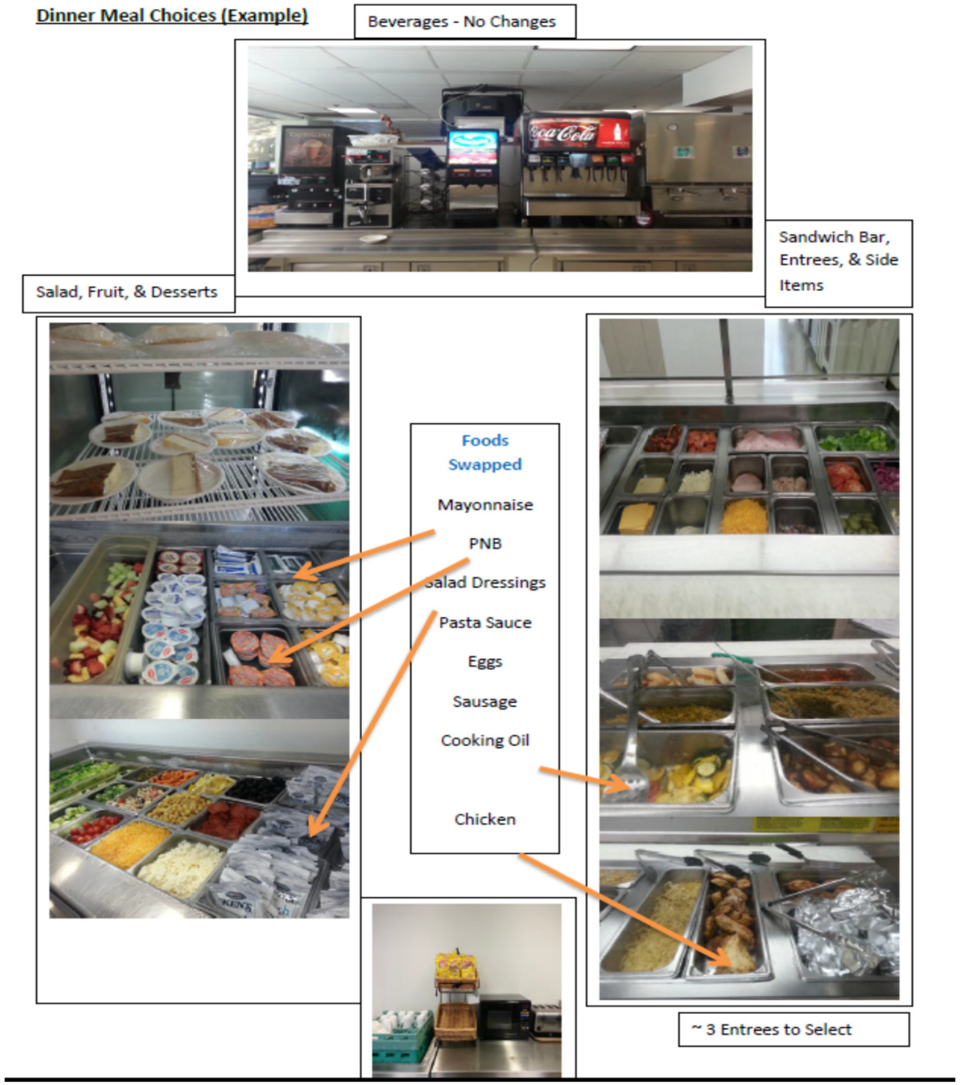

PNB= Peanut Butter

Supplementary Table S3. Study participants meals tracking form.

Nutritional Practices of Soldiers' Consuming Meals in Military Dining Facilities (14-14H)

APPENDIX D - DFAC Meals Tracking Form

MARKING INSTRUCTIONS	VOLUNTEER NUMBER	FILL IN TODAY'S DATE	
• Use a No. 2 pencil only.<br>• Do not use ink, ballpoint, or felt tip pens.<br>• Make solid marks / fill the response completely<br>• Erase cleanly any marks you wish to change.<br>• Make no stray marks on this form.<br>CORRECT: ● INCORRECT:	0 1 2 3 4 5 6 7 8 9<br>0 1 2 3 4 5 6 7 8 9<br>0 1 2 3 4 5 6 7 8 9	MONTH	0 1<br>0 1 2 3 4 5 6 7 8 9
		DAY	0 1 2 3<br>0 1 2 3 4 5 6 7 8 9
		YEAR 20__	0 1 2<br>0 1 2 3 4 5 6 7 8 9

Week # (example), filled in circle indicates meals consumed at the DFAC.

	SUNDAY	MONDAY	TUESDAY	WEDNESDAY	THURSDAY	FRIDAY	SATURDAY
A. Breakfast/Brunch	●	○	●	●	○	●	●
B. Lunch	●	●	●	●	●	●	○
C. Dinner/Supper	○	●	○	●	○	○	●

Goal: Please consume a minimum of 2 meals per day for 5 days a week duirng the 8-week study perios. Track only those meals consumed at the Natick DFAC. Thank you!

Week #1
Meal/Days

	SUNDAY	MONDAY	TUESDAY	WEDNESDAY	THURSDAY	FRIDAY	SATURDAY
A. Breakfast/Brunch	○	○	○	○	○	○	○
B. Lunch	○	○	○	○	○	○	○
C. Dinner/Supper	○	○	○	○	○	○	○

Week #2
Meal/Days

	SUNDAY	MONDAY	TUESDAY	WEDNESDAY	THURSDAY	FRIDAY	SATURDAY
A. Breakfast/Brunch	○	○	○	○	○	○	○
B. Lunch	○	○	○	○	○	○	○
C. Dinner/Supper	○	○	○	○	○	○	○

Week #3
Meal/Days

	SUNDAY	MONDAY	TUESDAY	WEDNESDAY	THURSDAY	FRIDAY	SATURDAY
A. Breakfast/Brunch	○	○	○	○	○	○	○
B. Lunch	○	○	○	○	○	○	○
C. Dinner/Supper	○	○	○	○	○	○	○

Nutritional Practices of Soldiers' Consuming Meals in Military Dining Facilities (14-14H)

Week #4

Meal/Days	SUNDAY	MONDAY	TUESDAY	WEDNESDAY	THURSDAY	FRIDAY	SATURDAY
A. Breakfast/Brunch							
B. Lunch							
C. Dinner/Supper							

Week #5

Meal/Days	SUNDAY	MONDAY	TUESDAY	WEDNESDAY	THURSDAY	FRIDAY	SATURDAY
A. Breakfast/Brunch							
B. Lunch							
C. Dinner/Supper							

Week #6

Meal/Days	SUNDAY	MONDAY	TUESDAY	WEDNESDAY	THURSDAY	FRIDAY	SATURDAY
A. Breakfast/Brunch							
B. Lunch							
C. Dinner/Supper							

Week #7

Meal/Days	SUNDAY	MONDAY	TUESDAY	WEDNESDAY	THURSDAY	FRIDAY	SATURDAY
A. Breakfast/Brunch							
B. Lunch							
C. Dinner/Supper							

Week #8

Meal/Days	SUNDAY	MONDAY	TUESDAY	WEDNESDAY	THURSDAY	FRIDAY	SATURDAY
A. Breakfast/Brunch							
B. Lunch							
C. Dinner/Supper							

[illegible]

Supplementary Table S4. Study participant food selection form

Nutritional Practices of Soldiers' Consuming Meals in Military Dining Facilities (14-14-H)

APPENDIX E - DFAC Food Selection Form

MARKING INSTRUCTIONS	VOLUNTEER NUMBER	FILL IN TODAY'S DATE
• Use a No. 2 pencil only.<br>• Do not use ink, ballpoint, or felt tip pens.<br>• Make solid marks / fill the response completely<br>• Erase cleanly any marks you wish to change.<br>• Make no stray marks on this form.	0 1 2 3 4 5 6 7 8 9<br>0 1 2 3 4 5 6 7 8 9<br>0 1 2 3 4 5 6 7 8 9	MONTH 0 1 / 0 1 2 3 4 5 6 7 8 9<br>DAY 0 1 2 3 / 0 1 2 3 4 5 6 7 8 9
CORRECT: ● INCORRECT: ⊘ ⊠ ◒ ◔	Week 1 2 3 4 5 6 7 8	YEAR 20__ 0 1 2 / 0 1 2 3 4 5 6 7 8 9

SUNDAY

BRUNCH	NUMBER OF SERVINGS	BRUNCH	NUMBER OF SERVINGS	DINNER	NUMBER OF SERVINGS
French toast	0 1 2 3 4	Chicken	0 1 2 3 4	Chicken	0 1 2 3 4
Pancakes	0 1 2 3 4	Fish	0 1 2 3 4	Fish	0 1 2 3 4
Eggs	0 1 2 3 4	Other meats	0 1 2 3 4	Other meats	0 1 2 3 4
Bacon	0 1 2 3 4	Vegetables	0 1 2 3 4	Vegetables	0 1 2 3 4
Sausage	0 1 2 3 4	Salad	0 1 2 3 4	Salad	0 1 2 3 4
Jam	0 1 2 3 4	Salad dressing	0 1 2 3 4	Salad dressing	0 1 2 3 4
Peanut butter	0 1 2 3 4	Peanut butter	0 1 2 3 4	Peanut butter	0 1 2 3 4
Fresh fruit	0 1 2 3 4	Mayonnaise	0 1 2 3 4	Mayonnaise	0 1 2 3 4
Milk	0 1 2 3 4	Pasta sauce	0 1 2 3 4	Pasta sauce	0 1 2 3 4

MONDAY

BREAKFAST	NUMBER OF SERVINGS	LUNCH	NUMBER OF SERVINGS	DINNER	NUMBER OF SERVINGS
French toast	0 1 2 3 4	Chicken	0 1 2 3 4	Chicken	0 1 2 3 4
Pancakes	0 1 2 3 4	Fish	0 1 2 3 4	Fish	0 1 2 3 4
Eggs	0 1 2 3 4	Other meats	0 1 2 3 4	Other meats	0 1 2 3 4
Bacon	0 1 2 3 4	Vegetables	0 1 2 3 4	Vegetables	0 1 2 3 4
Sausage	0 1 2 3 4	Salad	0 1 2 3 4	Salad	0 1 2 3 4
Jam	0 1 2 3 4	Salad dressing	0 1 2 3 4	Salad dressing	0 1 2 3 4
Peanut butter	0 1 2 3 4	Peanut butter	0 1 2 3 4	Peanut butter	0 1 2 3 4
Fresh fruit	0 1 2 3 4	Mayonnaise	0 1 2 3 4	Mayonnaise	0 1 2 3 4
Milk	0 1 2 3 4	Pasta sauce	0 1 2 3 4	Pasta sauce	0 1 2 3 4

TUESDAY

BREAKFAST	NUMBER OF SERVINGS	LUNCH	NUMBER OF SERVINGS	DINNER	NUMBER OF SERVINGS
French toast	0 1 2 3 4	Chicken	0 1 2 3 4	Chicken	0 1 2 3 4
Pancakes	0 1 2 3 4	Fish	0 1 2 3 4	Fish	0 1 2 3 4
Eggs	0 1 2 3 4	Other meats	0 1 2 3 4	Other meats	0 1 2 3 4
Bacon	0 1 2 3 4	Vegetables	0 1 2 3 4	Vegetables	0 1 2 3 4
Sausage	0 1 2 3 4	Salad	0 1 2 3 4	Salad	0 1 2 3 4
Jam	0 1 2 3 4	Salad dressing	0 1 2 3 4	Salad dressing	0 1 2 3 4
Peanut butter	0 1 2 3 4	Peanut butter	0 1 2 3 4	Peanut butter	0 1 2 3 4
Fresh fruit	0 1 2 3 4	Mayonnaise	0 1 2 3 4	Mayonnaise	0 1 2 3 4
Milk	0 1 2 3 4	Pasta sauce	0 1 2 3 4	Pasta sauce	0 1 2 3 4
